# Bachelors, Divorcees, and Widowers: Does Marriage Protect Men from Type 2 Diabetes?

**DOI:** 10.1371/journal.pone.0106720

**Published:** 2014-09-17

**Authors:** Marilyn C. Cornelis, Stephanie E. Chiuve, M. Maria Glymour, Shun-Chiao Chang, Eric J. Tchetgen Tchetgen, Liming Liang, Karestan C. Koenen, Eric B. Rimm, Ichiro Kawachi, Laura D. Kubzansky

**Affiliations:** 1 Department of Nutrition, Harvard School of Public Health, Boston, Massachusetts, United States of America; 2 Department of Epidemiology & Biostatistics, University of California San Francisco, San Francisco, California, United States of America; 3 Department of Social and Behavioral Sciences, Harvard School of Public Health, Boston, Massachusetts, United States of America; 4 Department of Epidemiology, Harvard School of Public Health, Boston, Massachusetts, United States of America; 5 Department of Epidemiology, Mailman School of Public Health, Columbia University, New York, New York, United States of America; University of Louisville, United States of America

## Abstract

While research has suggested that being married may confer a health advantage, few studies to date have investigated the role of marital status in the development of type 2 diabetes. We examined whether men who are not married have increased risk of incident type 2 diabetes in the Health Professionals Follow-up Study. Men (n = 41,378) who were free of T2D in 1986, were followed for ≤22 years with biennial reports of T2D, marital status and covariates. Cox proportional hazard models were used to compare risk of incident T2D by marital status (married vs unmarried and married vs never married, divorced/separated, or widowed). There were 2,952 cases of incident T2D. Compared to married men, unmarried men had a 16% higher risk of developing T2D (95%CI:1.04,1.30), adjusting for age, family history of diabetes, ethnicity, lifestyle and body mass index (BMI). Relative risks (RR) for developing T2D differed for divorced/separated (1.09 [95%CI: 0.94,1.27]), widowed (1.29 [95%CI:1.06,1.57]), and never married (1.17 [95%CI:0.91,1.52]) after adjusting for age, family history of diabetes and ethnicity. Adjusting for lifestyle and BMI, the RR for T2D associated with widowhood was no longer significant (RR:1.16 [95%CI:0.95,1.41]). When allowing for a 2-year lag period between marital status and disease, RRs of T2D for widowers were augmented and borderline significant (RR:1.24 [95%CI:1.00,1.54]) after full adjustment. In conclusion, not being married, and more specifically, widowhood was more consistently associated with an increased risk of type 2 diabetes in men and this may be mediated, in part, through unfavorable changes in lifestyle, diet and adiposity.

## Introduction

Diabetes is a leading cause of morbidity and mortality in developed countries. The worldwide prevalence of the disease is projected to double from the 371 million estimated in 2012 to 551 million in 2030; with type 2 diabetes (T2D) accounting for more than 90% of these cases [1]. Modifiable lifestyle factors such as adiposity, inactivity, smoking, excessive caloric intake and poor diet quality have consistently been associated with risk of T2D [2]. Such lifestyle risk factors are strongly influenced by social relationships, especially marriage, but the role of marital status in T2D risk has received remarkably little research attention.

Marriage is a common social relationship and key support mechanism for many adults, but the dissolution of marriage, either by widowhood or divorce is also common. Approximately 51% of American adults are currently married, while 6, 12, and 31% are widowed, divorced/separated or never-married, respectively [3]. Many health-enhancing properties of personal relationships, and particularly marriage, have been documented [4]. Married individuals may share a long-lasting supportive environment that enhances capacity to regulate and as a result fosters better physical and mental health than that of their unmarried counterparts [5], [6], [7]. Never entering marriage or marital termination by death or divorce has been shown to predict higher risk of premature mortality and cardiovascular disease, with more pronounced effects among men [8], [9], [10]. Four studies to date have investigated the role of marital status in the development of T2D. Three of these were small and cross-sectional [11], [12], [13]. In the only prospective study to date, marital status was not a significant predictor of incident T2D among obese men and women [14].

Understanding the effects of marital status on risk of incident T2D would inform health care providers about highly vulnerable populations, help design effective prevention interventions, and better elucidate the long-term health consequences of social ties. We therefore investigated whether men who are not currently married have increased risk of incident T2D in the Health Professionals Follow-up Study (HPFS), a large prospective cohort of men.

## Materials and Methods

### Study Population

The HPFS began in 1986 when 51, 529 male U.S. health professionals aged 40–75 answered a detailed questionnaire that included a comprehensive diet survey and items on lifestyle practice and medical history [15]. Cohort members are dentists, veterinarians, pharmacists, optometrists, osteopaths, and podiatrists. The cohort is followed through questionnaires mailed every other year, updating marital status and new medical diagnoses. Participants were excluded from analyses if, at baseline assessment, they provided no information on marital status (n = 411), or reported a history of type 2 (n = 250) or type 1 (n = 24) diabetes mellitus, cancer (n = 2047), cardiovascular disease (n = 3825), or stroke (n = 255). We further excluded participants with unknown date of death during follow-up and unknown type and date of diabetes diagnosis at baseline or during follow-up (n = 2100) as well as participants with missing baseline food frequency questionnaire (FFQ) (n = 1239). Therefore, 41,378 participants were included in the present analyses.

The study protocol was approved by the institutional review boards of Brigham and Women's Hospital and Harvard School of Public Health. The completion of self-administered questionnaires was considered to imply informed consent.

### Assessment of Type 2 Diabetes

Men with self-reported diagnoses of diabetes were mailed a supplementary questionnaire regarding symptoms, diagnostic tests, and hypoglycemic therapy. The validity of the supplementary questionnaire has been established through medical record review [16]. For cases before 1998, diagnosis was made using criteria proposed by the National Diabetes Data Group [17], which included one of the following: one or more classic symptoms (excessive thirst, polyuria, weight loss, hunger, pruritus, or coma) plus fasting plasma glucose ≥140 mg/dl (7.8 mmol/L) and/or random plasma glucose ≥200 mg/dl (11.1 mmol/L) and/or plasma glucose 2 hours after an oral glucose tolerance test ≥200 mg/dl; or at least two elevated plasma glucose levels on different occasions in the absence of symptoms; or treatment with hypoglycemic medication (insulin or oral hypoglycemic agent). Beginning in 1998, we used the American Diabetes Association's diagnostic criteria to diagnose diabetes cases [18]. These criteria were the same as those of the National Diabetes Data Group, except for the elevated fasting plasma glucose criterion for which the cut point was changed from 140 mg/dl to 126 mg/dl.

### Marital Status and Covariate Assessment

Marital status was reported every 2 years and covariates were reported every 2 to 4 years via standardized questionnaires. At each assessment, participants classified themselves as currently i) married, ii) never married, iii) divorced/separated, or iv) widowed. Body mass index (BMI) was calculated as weight (in kilograms) divided by the square of height (in meters). HPFS participants reported their average time engaged in eight specific physical activities (e.g., walking or hiking outdoors, running, bicycling) [19]. A metabolic equivalent task (MET)-hour score (MET-hours/week) was derived for each activity and then a total MET-hour score per week was calculated as the sum of MET values across all activities [20]. Self-administered questionnaires about body weight and physical activity have been previously validated in a sub-sample of this cohort [19], [21]. Biennial questionnaires assess cigarette smoking status (nonsmokers, past smokers, and current smokers). Ethnicity was assessed at baseline and a family history of diabetes (in first degree relatives) was assessed in a supplementary questionnaire administered 1987. A 131-item semi-quantitative FFQ was used to derive measures of daily nutrient intake. Detailed information regarding the development of the FFQ, procedures used to calculate daily energy-adjusted nutrient values, and reproducibility and validity of the questionnaire are documented elsewhere [22].

### Statistical Analysis

Men contributed person-time from the date of return of the 1986 questionnaire until incident T2D, death, or June 1, 2008, whichever came first. Age-adjusted general linear models were used for comparing means of covariates across marital status at baseline (1986). Cox proportional hazards models were first used to estimate age- and multivariable-adjusted relative risks (RRs) of developing T2D for unmarried versus married men. Further analyses were conducted comparing never married, divorced/separated or widowed men with married men, incorporating updated information on marital status over the course of the follow-up. For all analyses, the basic model included age (years), ethnicity (White, Asian, African American, other) and family history of diabetes (yes, no). Model 2 included covariates in the basic model plus lifestyle factors: smoking status (never, past, 1–14 cigs/day, 15–24 cigs/day, 25+ cigs/day), alcohol intake (<5 g/d, 5.0–9.9, 10.0–14.9, 15.0–29.9, or ≥30 g/day), multi-vitamin use (yes, no), and quintiles of physical activity (MET-hours/week), red/processed meats (servings/day), fruit (servings/day), vegetables (servings/day), glycemic load (g/day), trans fatty acid (g/day), cereal fiber (g/day), magnesium (mg/day) and calories/day. Model 3 included covariates in model 2 and BMI categories (<21, 21–22.9, 23–24.9, 25–26.9, 27–29.9, 30–32.9, 33–34.9, ≥35 kg/m^2^). All covariates are established risk factors for T2D and were associated with marital status in this cohort. However, lifestyle factors and BMI are also potential mediators, so effect estimates with adjustment for these pathway variables should be interpreted cautiously. We also considered models adjusted for living arrangement, ratio of polyunsaturated to saturated fatty acid intake, and consumption of whole grains, coffee, and sugar sweetened beverages. As there was little evidence of altered effect estimates when these factors were considered, they were not included in the final models.

To address the problem of missing values for marital status or covariates in the follow-up questionnaires, we replaced missing values with valid ones from a previous questionnaire. On average, 28% of HPFS participants had missing marital status on any of the follow-up biennial questionnaires. A comparison of men with complete marital status data in 1986 and 1990 but missing 1988, with men with complete data for 1986, 1988 and 1990, provided some reassurance that our replacement strategy was reasonable. Findings indicated similar proportions of men reporting a change in marital status between 1986 and 1990: 5.98% versus 5.16%. To better represent long-term diet and to minimize the within-person variation, we created cumulative averages of food and nutrient intake (per day) from baseline to the censoring events [23]. If participants reported new diagnoses of hypertension, hypercholesterolemia, cardiovascular disease, or cancer during follow-up, we carried forward the cumulative averages of dietary intake before disease onset to represent diet for later follow-up [23], [24]. To address potential time-varying confounding we also fitted marginal structural models (MSMs)[25]. We generated stabilized weights for MSMs based on the inverse of the probability of each man's marital status, given his past history of marital status and all covariates. Our MSM findings did not suggest significant time-varying confounding occurring after study enrollment. We therefore considered results synthesizing across the various Cox models as our primary findings because in the absence of time-varying confounding, the Cox models are more efficient than the MSM. We did not perform formal mediation analyses to evaluate potential mechanisms linking marital status and T2D, because we lack the data to assess whether there are unmeasured confounders of the hypothesized mediators and outcome (T2D). For example, adversity in childhood might lead to both higher likelihood of cigarette smoking and of T2D. Given that such unmeasured confounding is likely, we cannot fulfill the assumptions for mediation analyses. Such confounding biases estimates of mediation that are based on attenuation of effect estimates introduced by adjusting for the hypothesized mediator as compared to estimates in models that do not adjust for the hypothesized mediator [26]. Thus, it is important to note that attenuation of effect estimates in our analyses including measures of possible mediators may reflect some combination of partial mediation or bias due to unmeasured confounders of the hypothesized mediator and T2D [27].

To allow time for ‘status adaptation’, we analyzed the effect of latency time (time from exposure to T2D diagnosis) by relating each marital status assessment to T2D incidence 2 years after exposure. Statistical analyses were conducted using SAS software version 9 (SAS Institute Inc, Cary, North Carolina). All *p* values are 2-sided and a *p* value <0.05 is considered statistically significant.

## Results

Age-adjusted baseline characteristics of the cohort according to marital status are presented in Table 1. Married men (n = 37,625) were the least likely to be current cigarette smokers and multi-vitamin users. They consumed more red/processed meat, vegetables and cereal fiber than non-married men. Divorced/separated men (n = 2,352) consumed more alcohol, coffee and magnesium and were more likely to be current smokers but also engaged in more physical activity, compared to men in other marital arrangements. Widowers (n = 529) were older, more likely to report a family history of diabetes, consume more trans fat, and engage in the lowest levels of physical activity. Never married men (n = 872) were generally younger and leaner but they consumed a diet of higher glycemic load relative to current or previously married men.

**Table 1:** Age-Adjusted Baseline Characteristics of Men by Marital Status.

Characteristic		Married	Divorced/Separated	Widowed	Never married	*p Value* a
N		37,625	2,352	529	872	
Age, years (SD)		53.2 (9.5)	50.2 (8.2)	62.0 (8.6)	50.1 (9.2)	<.001
Ethnicity, %	European-white	95	95	93	93	
	Asian	2	1	2	3	
	African American	1	2	3	1	
	Other	2	2	2	3	
Family history of diabetes mellitus, %		13	12	12	14	
BMI, kg/m^2^ (SD)		24.9 (4.9)	24.5 (4.8)	25.0 (5.0)	24.2 (5.4)	<.001
Smoking status, %	Never	47	41	45	53	
	Past	41	39	36	30	
	Current	8	15	11	12	
Alcohol intake, %	0–4.9 g/day	48	38	45	51	
	5.0–29.9 g/day	41	43	42	37	
	30+ g/day	11	18	13	12	
Physical activity, MET-h/wk (SD)		21.1 (28.9)	25.0 (29.7)	20.5 (25.4)	21.2 (45.1)	<.001
Multivitamin-use, %		41	51	47	49	
Whole grain intake, g/d (SD)		21.8 (19.4)	22.5 (22.1)	21.6 (21.4)	21.2 (19.0)	0.2
Coffee intake, cups/d (SD)		1.9 (1.8)	2.0 (1.9)	2.0 (1.8)	1.7 (1.6)	<.001
Red/processed meat intake, servings/d (SD)		1.2 (0.8)	1.0 (0.8)	1.1 (0.8)	1.0 (0.8)	<.001
Fruit intake, serving/d (SD)		2.4 (1.6)	2.2 (1.8)	2.4 (2.1)	2.4 (1.8)	<.001
Vegetable intake, servings/d (SD)		3.1 (1.7)	2.8 (1.7)	2.8 (1.8)	2.9 (1.8)	<.001
Cereal fiber intake, g/d (SD)		5.9 (3.9)	5.6 (4.1)	5.6 (4.1)	5.7 (3.4)	<.001
Glycemic load, g/d (SD)		124 (26)	122 (28)	123 (27)	126 (28)	<.001
P:S intake, ratio)		0.6 (0.2)	0.6 (0.2)	0.6 (0.2)	0.6 (0.2)	0.71
Trans fatty-acid intake, g/d (SD)		1.3 (0.5)	1.2 (0.5)	1.3 (0.5)	1.3 (0.6)	<.001
Magnesium intake, mg/d (SD)		352 (82)	359 (90)	356 (91)	356 (94)	0.002

*Note.* SD = standard deviation; MET = metabolic equivalent task.

a Results from age-adjusted general linear models.

During 22 years of follow-up (801,807 person-years), we documented 2,952 new T2D diagnoses. Among men who were married at baseline, 14% reported a change in marital status during follow-up; likewise 61% of men who were divorced/separated at baseline, 50% of men who were widowers at baseline, and 20% of men who were never married at baseline, reported changes in marital status. Compared to married men, unmarried men had significantly increased incidence of T2D after adjusting for age, family history of diabetes and ethnicity (RR = 1.16, 95% confidence intervals [CI]: 1.04, 1.30); this association was unchanged when further adjusting for lifestyle risk factors and BMI (RR = 1.16 [1.04, 1.30]).

Important information may be lost using a simple definition of marital status. Thus, further analyses were conducted using more detailed marital status comparisons. In models adjusted for age, family history of diabetes and ethnicity, compared to married men, neither divorced/separated men (RR = 1.09 [0.94, 1.27]), nor never married men (RR = 1.17 [0.91, 1.52]) had significantly elevated risk of incident T2D. Widowed men, however, were at significantly elevated risk of T2D onset compared to married men (RR = 1.29 [1.06, 1.57]) (Table 2).

**Table 2:** Relative Risk (95% CI) of Incident Type 2 Diabetes According to Marital Status Between 1986 and 2008.

	Married	Div/Sep	Widowed	Never married
No.Cases/Person-years	2599/717393	185/48216	109/21521	59/14677
Incident rate/1000 person-years	3.62	3.84	5.06	4.02
Basic model^a^	Reference	1.09 (0.94,1.27)	1.29 (1.06,1.57)	1.17 (0.91,1.52)
Multivariable-adjusted^b^	Reference	1.12 (0.97,1.31)	1.21 (0.99,1.47)	1.18 (0.91,1.52)
Multivariable-adjusted + BMI^c^	Reference	1.14 (0.98,1.33)	1.16 (0.95,1.41)	1.24 (0.95,1.60)

a Adjusted for age (years), family history of diabetes, and ethnicity (White, Asian, African American, Other).

b Adjusted for terms in basic model and lifestyle factors: smoking status, alcohol intake, multi-vitamin use, physical activity, red/processed meats, fruit, vegetables, glycemic load, trans fatty acid, cereal fiber, magnesium and calories/day.

c Further adjusted for eight BMI categories.

Considering the effect of including potential pathways factors in the model, the RR of T2D associated with widowhood was slightly attenuated after adjusting for lifestyle factors (RR = 1.21 [0.99,1.47]) and was no longer significant when further adjusting for BMI (RR = 1.16 [0.95,1.41]). The opposite trend was observed for divorced/separated men: adjustment for potential pathway factors of lifestyle (RR = 1.12 [0.97, 1.31]) as well as BMI (RR = 1.14 [0.98, 1.33]) increased the point estimate for the association between divorce/separated and T2D compared to the minimally adjusted effect estimate (1.09). After adjusting for all pathway factors and specifically BMI there was an enhanced risk of T2D among never married men (RR = 1.24 [0.95, 1.60]); although this did not reach statistical significance. Similar results were observed when excluding smokers; a lifestyle factor previously associated with beneficial changes in BMI in this cohort [28].

Results from MSMs had wide CIs that included point estimates from all three of the conventional models, with the exception of the estimate for never married men. When applying stabilized weights derived from all covariates (i.e. multivariable-adjusted + BMI in Table 2), RRs (95% CI) for developing T2D among divorced/separated, widowed and never married men were 1.48 (0.89, 2.46), 0.96 (0.65, 1.40), and 2.98 (1.61, 5.53), respectively. We also examined marital status incorporating a 2-year lag period between exposure and outcome. Risk associations with widowhood were augmented and remained significant, even after adjusting for all potential confounders and pathway variables (RR = 1.24 [1.00, 1.55]) (Table 3). Similar to analyses reported above, divorced/separated or never married men were not at a strongly increased risk of T2D compared to married men.

**Table 3:** Relative Risk (95% CI) of Incident Type 2 Diabetes According to Marital Status with 2-Year Exposure Lag.

	Married	Div/Sep	Widowed	Never married
No.Cases/Person-years	2372/640106	172/43045	88/16509	57/13258
Incident rate/1000 person-years	3.71	4.00	5.33	4.30
Basic model^a^	Reference	1.10 (0.94,1.28)	1.39 (1.12,1.73)	1.21 (0.93,1.57)
Multivariable-adjusted^b^	Reference	1.13 (0.97,1.32)	1.31 (1.05,1.63)	1.22 (0.93,1.58)
Multivariable-adjusted + BMI^c^	Reference	1.15 (0.97,1.35)	1.24 (1.00,1.54)	1.28 (0.98,1.67)

a Adjusted for age (years), family history of diabetes, and ethnicity (White, Asian, African American, Other)

b Adjusted for terms in basic model and lifestyle factors: smoking status, alcohol intake, multi-vitamin use, physical activity, red/processed meats, fruit, vegetables, glycemic load, trans fatty acid, cereal fiber, magnesium and calories/day.

c Further adjusted for eight BMI categories.

## Discussion

We examined risk of incident T2D associated with current marital status in a prospective analysis of male health professionals. After 22 years of follow-up, we observed a significantly increased risk of T2D among unmarried men. Using a more nuanced assessment of marital status suggested that widowers in particular were at elevated risk of T2D. The association between widowhood and T2D was attenuated with the inclusion of lifestyle factors and BMI; these variables are often hypothesized to serve as pathways linking social ties and health [29]. Effects were strengthened by incorporating time for potential effects to become manifest: focusing on diabetes risk two years after marital status assessment resulted in even stronger associations.

Our findings are consistent with much prior evidence on the cardiovascular health effects of marriage, but extend these results substantially by providing evidence on T2D and contrasting risk among bachelors, divorcees, and widowers compared to married men. A “widowhood” or “bereavement” effect has been demonstrated in numerous studies of mortality [10]. Loss of a spouse has been associated with more depressive symptoms, poorer physical and cognitive function, worse self-rated health, increased risk of institutionalization [8], as well as acute myocardial infarction [30]. Two earlier cross-sectional studies reported a higher prevalence of diabetes among the widowed compared to married individuals [12], [13]. Diabetes was less common among married compared to unmarried, widowed or divorced subjects in a cross-sectional analysis of 379 non-institutionalized men and women ages 70 years or over [11]. Friedrich *et al* [14] explored correlates of adverse outcomes in 1,506 abdominally obese men and women in the population-based Study of Health in Pomerani. Baseline marital status was defined as one of three categories: i) married, ii) single, or iii) divorced/widowed, and was not a significant predictor of T2D five years later. The large sample size, detailed information on marital status and long follow-up in an initially healthy population are key properties which distinguish the current study from previous studies of marital status and T2D.

The two major hypotheses for morbidity and mortality advantages of *married* individuals are ‘selection’ (i.e., healthier individuals are more likely to marry and remain married), and ‘protection’ (i.e., marriage provides resources, reduces stress, loneliness, and risky health habits, and thereby improves long term health outcomes) [5], [31], [32]. The size and design of the current study allowed us to address both arguments in the context of T2D risk for the first time. In minimally adjusted models, widowers were at elevated risk of T2D, suggesting the straightforward notion of ‘selection,’ — that healthier individuals are more likely to marry — is insufficient to explain the health advantage of married individuals. T2D risk associated with widowhood was attenuated when accounting for lifestyle factors and BMI, while risk associated with bachelorhood was generally strengthened with adjustment for these covariates; lending some support for the ‘protection’ theory. However, our findings of greater risk among widowers relative to those who were divorced (particularly in the lagged analyses) suggest that the relationship may not be mediated by the protective effect of marriage or even aversive effects of marriage dissolution, *per se*. Rather, some stressor, whether it be environmental or psychosocial, experienced with spousal bereavement appears distinct from stresses arising from divorce. Both widowhood and divorce are stressful life events, but they may affect lifestyle behaviors predisposing to T2D development differently [29]. For example, divorce may be a mutual and foreseeable process, marking the termination of an unsatisfying marriage, whereas spousal death is usually unwelcome and beyond the control of the surviving spouse. In our sample, the recently widowed were also less likely to re-marry. The immediate and future health consequences of these two marital statuses may therefore be distinct.

In our population of health professionals, response to bereavement may have included unfavorable changes in health behavior which increase risk for T2D. The strengthening of associations in lagged analyses suggests such effects may become more pronounced over time. If this result is confirmed, it is an important insight into the health effects of widowhood, because other research has demonstrated acute effects of bereavement on mortality and myocardial infarction immediately following the death of a spouse [10], [30]. Widowhood may thus have both acute and chronic, long-term effects. For individuals who survive the “high risk” period, there may be increased health risks conferred by other pathways related to behavioral mechanisms.

Although we observed no significant risk of T2D among divorcees or bachelors in minimally adjusted models, effect estimates tended to increase when adjusting for lifestyle factors. This is consistent with some previous studies, which suggest divorce may be associated with weight loss, whereas marriage is associated with weight gain, overweight and reduced fitness levels [5], [33], [34], [35], [36]. Bachelorhood may represent a unique risk category, with some beneficial consequences for weight-related risk factors but adverse effects via other mechanisms. Mediation analyses with detailed time-varying assessments are necessary to disentangle these complex, dynamic relationships.

Strengths of the current study include its large sample size, prospective design, long follow-up and availability of repeated measures of marital status and potential confounders and/or mediating factors. Moreover, consistent (and stronger) associations found in the lagged analyses provides reassurance that effects are less likely due to incipient disease processes present at the time of widowhood. Nevertheless, several limitations should be considered. Some of the control participants may have undiagnosed T2D that would bias the results toward the null. However, in a previous validation study [37], the prevalence of undiagnosed T2D in this sample of health professionals was quite low (∼2%) and substantially lower than that in the general population (∼30%)[38]. The marital status and risk factor profiles of HPFS participants may differ from those of women and other populations, thus limiting the generalizability of our results. This is especially salient because the behavioral consequences of marriage probably differ for men and women. Information on many key plausible mediators, e.g., depression was not collected. We used marital status as a proxy measure for spousal support and control recognizing that non-spousal cohabitating partners may provide similar support and control to unmarried men, as well as engage in other types of spousal interactions that are not aimed at promoting healthful behavior [7]. We had no information on marital quality. Insufficient information on timing of widowhood, particularly prior to baseline (1986), also limited our ability to study the impact of time since loss of spouse on T2D risk.

In conclusion, widowed men had increased risk of T2D and this may be mediated, in part, through unfavorable changes in lifestyle, diet and adiposity. Bachelors do not have, on net, significant elevations in T2D compared to married men, but this may be due to (unmeasured) protective factors that offset any increased risk attributable to other factors. There is little evidence for elevated T2D risk among men who were divorced or separated. Our findings, together with prior work showing early mortality and elevated CHD among widows, underscore the need for closer attention to this older, and thus already especially vulnerable, population. Death of a spouse currently ranks as the life-event needing the most intense *social* readjustment [8]. Physicians should also be aware of possible long term health risks emerging after widowhood, which may be remedied by attention to healthy behaviors. Overall, more awareness of the social arrangements in patient's lives may aid physicians' ability to implement timely preventive or intervention strategies.
